# Forebrain Neural Precursor Cells Are Differentially Vulnerable to Zika Virus Infection

**DOI:** 10.1523/ENEURO.0108-21.2021

**Published:** 2021-09-08

**Authors:** Samantha M. Shelton, Alexandra R. Soucy, Ronni Kurzion, Ella Zeldich, John H. Connor, Tarik F. Haydar

**Affiliations:** 1 Department of Anatomy and Neurobiology, Boston University School of Medicine, Boston, MA 02118; 2 Graduate Program in Neuroscience, Boston University School of Medicine, Boston, MA 02118; 3 Department of Microbiology and National Emerging Infectious Diseases Laboratories, Boston University Medical Campus, Boston, MA 02118; 4 Center for Neuroscience Research, Children’s National Hospital, Washington, DC 20010

**Keywords:** corticogenesis, development, heterogeneity, microcephaly, radial glia, Zika

## Abstract

Prenatal exposure to Zika virus (ZIKV) can result in microencephaly and congenital Zika syndrome, although some brain cells and structures are spared by the virus for unknown reasons. Here, a novel murine model of fetal ZIKV infection incorporating intraventricular infection and cell type-specific *in utero* electroporation (IUE) was used to identify the time course of ZIKV infection and to determine the identity of cells that are initially infected or spared during neocortical neurogenesis. *In vivo* time course studies revealed the presence of ZIKV in apical radial glial cells (aRGCs) at early time points following virus exposure, while basal intermediate progenitor cells (bIPCs) became maximally (ZIKV^+^) after 3 d of virus exposure. ZIKV-infected fetal brains exhibited microencephaly as early as 1 d following infection, regardless of developmental age. This change in brain size was caused in part by apoptosis and reduced proliferation that persisted until birth. While 60% of aRGC basal fibers were perturbed during infection, 40% retained normal morphology, indicating that aRGCs are not uniformly vulnerable to ZIKV infection. To investigate this heterogeneous vulnerability, we performed genetic fate mapping using cell type-specific probes derived from a mouse embryonic day (E)15.5 neocortical wall single-cell RNA sequencing (scRNAseq) dataset. The results indicate that one class of aRGCs preferentially express the putative ZIKV entry receptor *AXL* and that these cells are more vulnerable to ZIKV infection than other aRGC subtypes with low *AXL* expression. Together, these data uncover crucial temporal and cellular details of ZIKV fetal brain infection for prevention strategies and for management of congenital Zika syndrome.

## Significance Statement

The transcriptional signatures of neural precursor cells (NPCs) were used for the first time to test Zika virus (ZIKV) susceptibility in a direct fetal brain infection model. This novel methodology allowed for elucidation of time point-specific differences in NPC susceptibility that have been debated in the field. Additionally, elucidation of cell morphologic features using *in utero* electroporation (IUE) revealed substantial but incomplete interruption of basal fibers, a finding that implies interference with neuronal migration. The model presented here, allows for assessment of prenatal development after exposure to a variety of viruses. The improved specificity of apical radial glial cell (aRGC) labeling afforded by the cell-specific labeling tools uncover functional differences between aRGC types that will have important implications for children exposed to ZIKV as well as for understanding corticogenesis.

## Introduction

The 2015 Brazilian Zika virus (ZIKV) outbreak has been associated with an estimated 20-fold increase in cases of microcephaly in newborns (Center for Disease Control, 2017). Despite the strong association between prenatal ZIKV exposure and the devastating effects of microcephaly, there are no available treatments or approved vaccines for ZIKV despite the rapid response by the biomedical community (CDC, 2019). Greater understanding of the mechanisms underlying ZIKV-induced microcephaly is needed to aid in the development of therapeutic strategies. In particular, the infection route and cellular mechanisms contributing to ZIKV-induced microcephaly are still unclear. The possibility that cells are not uniformly affected by virus exposure may lead to new therapies or prevention strategies.

Various approaches have been used to understand the mechanisms behind ZIKV-induced microencephaly, including cell culture, organoids, slice culture, and animal models (Haubensak et al., 2004; Bhattacharyya et al., 2013; Gadea et al., 2016; Lazear et al., 2016; Miner and Diamond, 2016; Nowakowski et al., 2016; Onorati et al., 2016; Shao et al., 2016; Tang et al., 2016; Dudley et al., 2017; Meertens et al., 2017; Rosenfeld et al., 2017; Vanwalscappel et al., 2018; Politi et al., 2020). Major obstacles for model development have included the differences in immune responses between humans and rodents. While ZIKV exhibits tropism in human maternal and fetal epithelium during gestation, the maternal immune system and placental barrier in mice prevents fetal infection (Lazear et al., 2016). Interferon knock-out mice allow maternal-fetal transmission of ZIKV, thus serving as a potential system of study (Lazear et al., 2016). A challenge of this model is that the loss of interferon likely alters the natural progression of infection, cell death, and proliferation in the central nervous system, impairing the translatability of findings from these models (Bhattacharyya et al., 2013; Meertens et al., 2017; Vanwalscappel et al., 2018). Studies using 1D, 2D, and 3D stem cell cultures have demonstrated key aspects of ZIKV infection, including increased cell death and decreased proliferation, but these methods do not contain the full repertoire of cell types or developmental processes necessary for proper brain development (Gadea et al., 2016; Miner and Diamond, 2016; Tang et al., 2016; Meertens et al., 2017). Similarly, while assessments using *in utero* electroporation (IUE) have identified morphologic changes to precursors and neurons in response to ZIKV, they have not reached cell type-specific resolution (Rosenfeld et al., 2017). Several other studies have been performed with inactivated ZIKV or with one of the first available strains of the virus, MR-766 (Gadea et al., 2016; Tang et al., 2016). Conclusions from these studies exhibited lack of viral transmission and attenuating mutations within research stocks of MR-766. To overcome these obstacles, we sought to improve on the IUE infection model that circumvents the dam’s immune defenses, permitting study of infection from a contemporary strain of ZIKV over the period of cortical development *in utero.*

**Figure 1.**
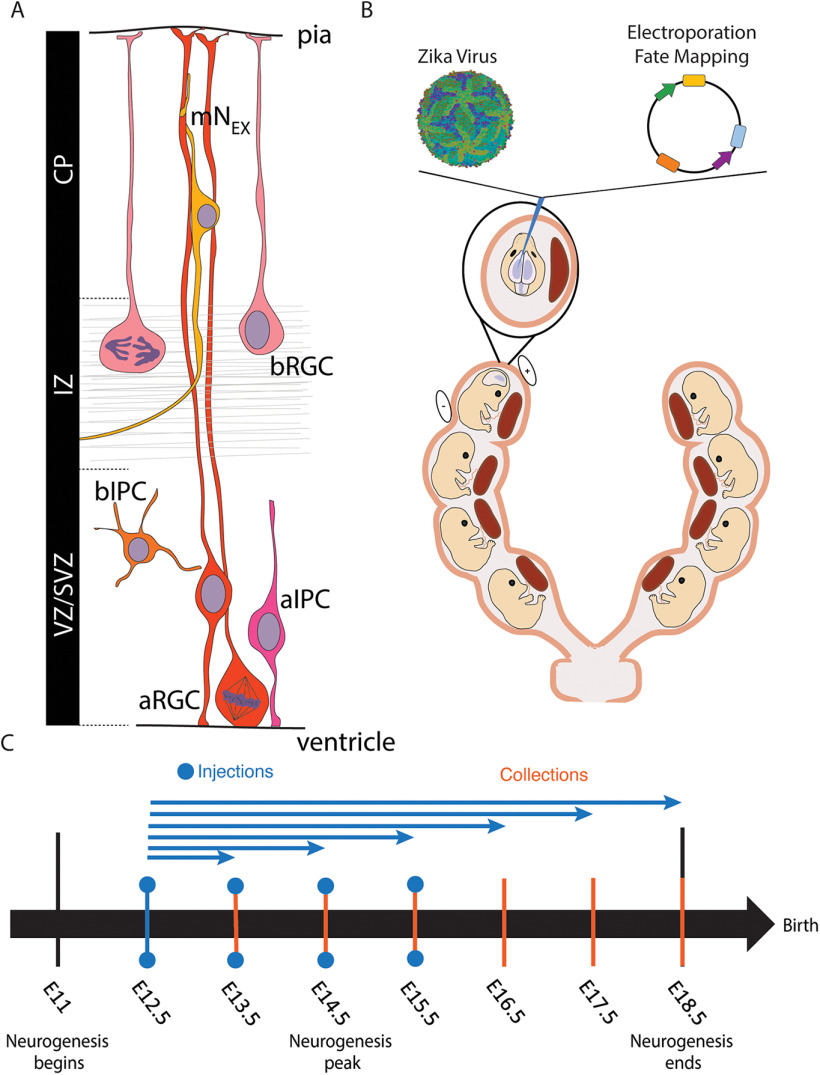
Intraventricular ZIKV model and genetic fate mapping approach. ***A***, Each NPC of the cerebral cortex has a unique transcriptional profile, morphology, and location of mitosis. ***B***, A total of 10,000 FFU ZIKV or saline, and cell type-specific plasmid DNA are injected into the lateral ventricle. ***C***, IUE and ZIKV infection experiments include collection on E13.5–E18.5 encompassing early, mid, and late neurogenesis. The blue arrows denote the temporal infection-collection plan for the E12.5 experiments for example.

We have combined this direct brain infection model with cell type-specific methods to determine which groups of neural precursor cells (NPCs) are most susceptible to prenatal ZIKV exposure. In the mouse neocortex, all lineages of neurons are born between embryonic day (E)11 and E17 from a diverse population of precursor cell types. These neurons are generated either directly or indirectly from a secondary progenitor. This period of cortical expansion is essential to brain development, and the ratio of direct and indirect neurogenesis has a large impact on the eventual size and neuronal complexity of the neocortex (Rakic, 1987, 1988, 1995). Thus, viral effects on individual NPC populations could result in smaller populations of their specific neuronal progeny, resulting in microencephaly and dysgenesis of particular cell populations in the cortical architecture. Previous studies have shown that apical radial glial cells (aRGCs) are preferentially targeted by ZIKV while others have suggested that basal intermediate progenitor cells (bIPCs) are the target of infection (Onorati et al., 2016; Lin et al., 2017). Emerging data on differences between progenitor cell groups might be crucial in elucidating nuanced mechanisms of ZIKV infection and consequent changes in neuronal output. NPCs can be categorized into sub-classes by transcriptional profile, morphology, and location within the ventricular zone (VZ) and sub-VZ (SVZ; Fig. 1*A*; Rakic, 1972; Hartfuss et al., 2001; Tamamaki et al., 2001; Holsapple et al., 2003; Haubensak et al., 2004; Miyata et al., 2004; Noctor et al., 2004; Englund et al., 2005; Tyler et al., 2015; Tasic et al., 2018; Hodge et al., 2019; Heavner et al., 2020; Li et al., 2020). Recent advancements in single-cell RNA sequencing (scRNAseq) provide the opportunity to study the heterogeneity of NPCs and their daughter cells at new levels of resolution. We sought to make use of these transcriptomic distinctions between NPCs to determine the cell type-specific infection profile of ZIKV over time *in vivo*.

We therefore developed a method to directly infect the telencephalon of a non-immunocompromised mouse model with a contemporary strain and physiological titer of active ZIKV with low passage number. To study subtypes of NPCs in the infected developing brain, we performed IUE during ZIKV exposure (Fig. 1*B*). This technique enabled us to study the infection process in apical precursors (aRGCs, apical IPCs) and basal precursors (bIPCs and basal RGCs) over time *in vivo* (Tyler et al., 2015). These findings demonstrate the effective use of a novel *in vivo* method for studying the effects of ZIKV infection on the developing brain, and they provide new insight into corticogenesis in children affected by virus-induced microencephaly.

## Materials and Methods

All materials and supplies are listed in Table 2 and all statistical results are presented in Table 3.

### ZIKV production

Vero E6 cells were maintained and passaged in cMEM [MEM (Invitrogen) containing 10% fetal bovine serum (Sigma Life Science)]. The ZIKV PRVABC59 (2015 Puerto Rico strain, GenBank KZ087101.2) was obtained from BEI resources. Master stocks were prepared from supernatants of Vero E6 cells infected at low MOI as published in Rossignol et al. (2017).

### Animals and IUE

IUE was performed on timed pregnant CD-1 dams purchased from Charles River Laboratories. Dams were separated into individual cages on arrival to prevent over-crowding. Animals were allowed to rest for 72 h in standard cages with huts and a ZT12. Dams were anesthetized via intraperitoneal injection of a ketamine/xylazine mixture (10 mg/kg), and the uterine horns were exposed by midline laparotomy. One to two microliters of plasmid DNA mixed with 0.1% fast green dye (Sigma-Aldrich) was injected intracerebrally, through the uterine wall and amniotic sac, via a pulled glass micropipette. Cre and reporter plasmid DNA was mixed at a 1:1 ratio by copy number, and the final concentration of each plasmid was between 2 and 3 μg/μl. The anode of a Tweezertrode (Harvard Apparatus) was placed over the dorsal telencephalon above the uterine muscle, and four 35-V pulses (50-ms duration separated by a 950-ms interval) were applied with a BTX ECM830 pulse generator (Harvard Apparatus). Following electroporation, the uterine horns were returned to the abdomen and the cavity was filled with a warm 0.9% sterile saline solution. The incisions were closed with silk or absorbable sutures depending on the duration of the experiment. Dams were then placed in a clean cage and monitored closely during recovery. After either 24 or 48 h mice were killed with CO_2_ asphyxiation and cervical dislocation. Male and female pups were removed from the uterus and decapitated before drop fixation. All animal procedures were performed in accordance with the Boston University animal care committee’s regulations.

To model infection of a contemporary ZIKV in fetal brain, we chose to directly inject the PRVAB59 strain into the lateral ventricle of prenatal mice *in utero*. The concentration was determined after initial tests of infectability at concentrations ranging from 1000 to 12,000 FFU. We used the lowest concentration able to cause reliable infection to better model the expected viral load that would be transmitted by infected mothers to human fetuses. The average bolus from the *Aedes aegypti* mosquito is 10^4^−10^8^ FFU. Some of that bolus is cleared from the maternal blood stream before infecting a fetus, making the expected titer around 10^1–3^ FFU (Dudley et al., 2017). 10^3^ FFU was sufficient to cause infection in mice without causing large scale cell death. Antibodies to ZIKV envelope protein resulted in false positives in western blot, therefore antibody against non-structural protein 2b (NS2B) was used for all experiments. To infect embryos at the time of IUE, 10^3^ FFU was added to the plasmid DNA and fast green dye cocktail in place of saline. Embryos in one uterine horn were injected with plasmid DNA cocktail containing virus and embryos in the contralateral uterine horn were injected with plasmid DNA cocktail containing saline to provide an age-matched vehicle control. Immunohistochemistry (IHC) against ZIKV and RT-qPCR were conducted to ensure that ZIKV did not infect the control embryos. These procedures were reviewed and approved by the Institutional Animal Care and Use Committee at the Boston University.

### Cloning

The cloning into the recipient Cre recombinase vector was done using In-Fusion technology and the backbone vector carrying Cre was amplified using primers forward, CTCGAGGGGCAGAGCC; reverse, GGTACCCAATTCGCCCTATAGTGAG (Tyler and Haydar, 2013).

A 2.9-kb fragment of DNA upstream of the *Prc1* translation initiation (Li et al., 2004) site was amplified by PCR from mouse genomic DNA with CloneAmp HiFi PCR Premix (TakaraBio) using the following primers with the added 15-bp overlap at their ends: forward, TACCGTTCTCCGTCCCGCTCGAGGGGCAGAGCCG; reverse, GCTCTGCCCCTCGAGCGGGACGGAGAACGG; forward, GGCGAATTGGGTACCCTTGGCTTGCTAGGGTGTGA; reverse, CCCTAGCAAGCCAAGCGCTATC). The 3.2-kb *Robo4* promoter (Okada et al., 2015) was amplified with the same overlapping ends using forward, GGCGAATTGGGTACCCATGCATTTGGAGTTTCCATGTCCT and reverse, GCTCTGCCCCTCGAGGGCTGCTCTCGGCTCC. The 424-bp *MFAP2* promoter (*add 16321658*) was amplified the same way using primers forward, GGCGAATTGGGTACCACTCGATCTCCCTTAATCTGCCT; reverse, GGCGAATTGGGTACCACTCGATCTCCCTTAATCTGCCT.

Amplified DNA for the inserts and the vector was purified with a NucleoSpin PCR Clean-UP kit (TakaraBio) and 25–50 ng of DNA were fused together using In-Fusion HD enzyme premix (TakaraBio). The fused product was transformed into chemically competent Stellar competent cells (TakaraBio) for selection by growth on LB Amp agar plates. Cloning was confirmed by DNA sequencing.

### RT-qPCR

RNA was extracted from flash frozen tissue with a QIAGEN RNeasy kit per manufacturer recommendations. Quantitative RT-qPCR was conducted with a Roche one-step SYBR ZsGreen kit. Primers used were ZIKV forward, AARTACACATACCARAACAAAGTGGT and ZIKV reverse, TCCRCTCCCYCTYTGGTCTTG.

### Lipophilic staining

DiI (1 mg/ml) was dissolved in 100% ethanol and allowed to air dry on a Petri dish until crystals formed (∼1 h). Meninges were removed from brains that were fixed in 4% paraformaldehyde (PFA) overnight. The dorsal surface of the brain was rolled over the bed of DiI crystals to create a uniform coating. The labeled brains were stored in 0.02% sodium azide in PBS, light protected at room temperature for two weeks. Brains were mounted in 4% agarose and cut into 80-μm sections with a Leica VT1000S vibrating microtome; 75-μm Z-stack images were taken using an upright Zeiss LSM 710 microscope. Cells were manually scored and counted using the LSM image browser software.

### IHC

Embryos were collected at 1–6 d postinfection (DPI), and the heads were fixed overnight in 4% PFA, cryoprotected in 30% sucrose for 24–48 h, and frozen in OCT compound in tissue molds with an ethanol/dry ice bath. Frozen tissue was cut into 16-μm sections using a HM 550 Cryostar cryostat and mounted and dried onto superfrost slides.

Before staining, frozen sections were air dried for 1 h and washed three times in PBS for 5 min each. Sections were then blocked in diluent (5% goat serum, 0.3% Triton X-100, 1× PBS) for 1 h at room temperature.

Incubation with primary antibodies, mouse anti-Ki67 (1:250, Thermofisher), rabbit anti-phospho-histone H3 (PH3; 1:300, Millipore), rabbit-Tbr2 (1:500, Millipore), goat anti-Sox2 (1:250, Santa Cruz), rabbit-NS2B (1:250, Genetex), and rabbit anti-cleaved caspase 3 (CC3; 1:250, Cell Signaling) were performed overnight at room temperature. Following three 5-min washes in PBS, sections were incubated for 2 h at room temperature in diluent containing the appropriate Alexa Fluor-conjugated secondary antibodies (1:500 for all). Sections were washed an additional three times for 5 min and stained with Hoechst at 1:1000 in PBS for 10 min. Slides were washed with 1× PBS three times for 5 min and coverslipped with fluoromount-G. The 20× *Z*-stack images (20 μm) were acquired using an upright Zeiss LSM 710 microscope and positive cells from *n* =* *4 brains were identified and counted using LSM image browser software.

ZsGreen^+^ and mCherry^+^ cells from electroporation were manually scored and counted using the LSM image browser software. Cells were classified as “ZsGreen^+^” if they expressed ZsGreen but contained no signal for mCherry. Correspondingly, cells were classified “mCherry^+^” if they contained mCherry signal, whether or not they were also ZsGreen^+^ because of perdurance of the fluorescent protein, remaining copies of unrecombined reporter plasmid, or because of the presence of a subpopulation of precursors transitioning from an apical to a basal fate.

### Fluorescent *in situ* hybridization

Fluorescent *in situ* hybridization was performed using the RNAscope (ACDbio) protocol for fixed frozen sections with the modification of using 100% methanol with 3% hydrogen peroxide rather than using 100% peroxide to preserve the integrity of the tissue. The protocol can be found on the ACDbio webpage (https://acdbio.com/technical-support/user-manuals) under user manual document number 320293-USM. Cells were counted as being positive if they had two or more puncta co-localized to the nucleus labeled with Hoechst. The 20× *Z*-stack images (20 μm) were acquired using an upright Zeiss LSM 710 microscope and positive cells from *n *=* *4 brains were identified and counted using LSM image browser software.

### Analysis of differentially expressed genes from scRNAseq data

Genes that had high expression in only one group and that were expressed in the majority of cells within a group were then assessed for location of gene expression. The online Allen Brain Atlas was used to investigate *in situ* hybridization of genes of interest to verify expression in the VZ of developing mouse brain (Allen Brain Developing Mouse Brain Atlas, http://developingmouse.brain-map.org). From there, a short list was generated of genes that showed a significant difference in gene expression between aRGC groups 1 and 2, that had high expression in many cells within their cluster and showed gene expression localized to the VZ of the developing murine cortex. Markers who showed expression in cell types other than aRGCs and that were expressed outside of the VZ at E15.5 were excluded. Additionally, the find markers algorithm in the Seurat package offered in R was used to corroborate this list. Only the two most specific markers were used for each aRGC group.

### Statistics

A two-way Student’s *t* test was run for the log fold change of each gene in aRGC groups 1 and 2 to compare differential gene expression between the two groups. Histograms of *z* scores were generated for genes that reached significance. Genes that had high expression in only one group and that were expressed in the majority of cells within a group were then assessed for location of gene expression. These top candidate markers were secondarily validated with the Seurat Find Markers algorithm.

Cell counts were analyzed in Microsoft excel and Prism using a two-way Student’s *t* tests with a *p* < 0.05 considered statistically significant when comparing control and infected samples. When comparing statistical differences among the means of two or more groups, a one-way ANOVA was performed in Prism with *p* < 0.05 considered statistically significant.

## Results

### Active ZIKV injection into the lateral ventricle of the prenatal mouse brain

**Figure 2.**
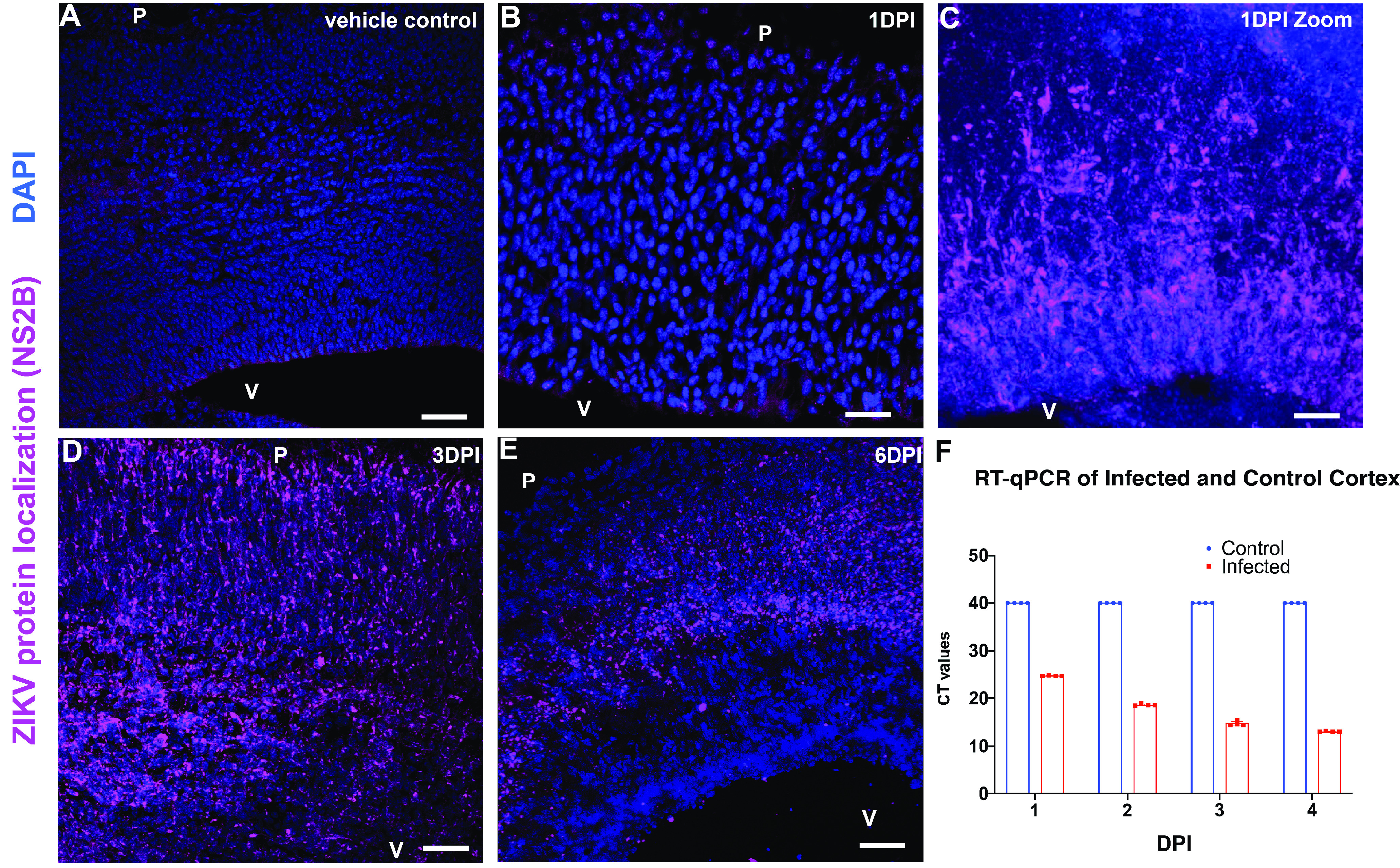
Fetal ZIKV infection spreads radially throughout the telencephalon. NS2B immunofluorescence in fetal brains infected intraventricularly on E12.5 with 10,000 FFU of ZIKV and collected 1, 3, and 6 DPI. ***A***, Vehicle control neocortical wall at 3 DPI. Scale bar: 60 μm. ***B***, Infected neocortical wall at 1 DPI. Scale bar: 30 μm. ***C***, High-magnification image of infected neocortical wall at 1 DPI. Scale bar: 10 μm. ***D***, Infected neocortical wall at 3 DPI. Scale bar: 60 μm. ***E***, Infected neocortical wall at 6 DPI. Scale bar: 60 μm. ***F***, Presence of ZIKV was also measured with RT-qPCR in vehicle-injected and ZIKV-injected fetal brains at 1, 2, 3, and 4 DPI. The vehicle-injected brains had RT-qPCR cycle thresholds (CTs) at or above 40 cycles, indicating no presence of virus. The number of amplification cycles necessary to detect ZIKV decreased with each day after injection (*n* = 4, 4).

To model infection of a contemporary ZIKV strain in the fetal brain, 10,000 FFU of the PRVAB59 strain was injected into the lateral ventricle of prenatal mice *in utero*. This concentration was determined after initial tests of infectability within a 5000–12,000 FFU range. We used the lowest concentration necessary to cause reliable infection to better model the expected viral load that would be transmitted by infected mothers to human fetuses. The average bolus from the *A. aegypti* mosquito is 10^1–3^ FFU (Dudley et al., 2017). We found that 10^3^ FFU was sufficient to cause reliable infection in the fetal mouse brain. IHC for NS2B was used to test ZIKV viral replication for all experiments. IHC using NS2B was employed to test for the presence of replicating ZIKV in control and infected embryos at various times following infection (Fig. 2). IHC (Fig. 2*A–E*) and RT-qPCR (Fig. 2*F*) studies were conducted to determine that the virus did not pass across the cervix to cause infection in the contralateral uterine horn, which was used for age-matched saline vehicle controls. Widespread infection was low at 1 DPI and steadily increased in the following days. By 6 DPI, most virus was present in the cortical neuron layers in the VZ, indicating that ZIKV had been cleared from the cerebral spinal fluid of the lateral ventricles and was replicating in cells located away from the ventricle by this time point (Fig. 2*E*).

### Time course of ZIKV-induced microencephaly

**Figure 3.**
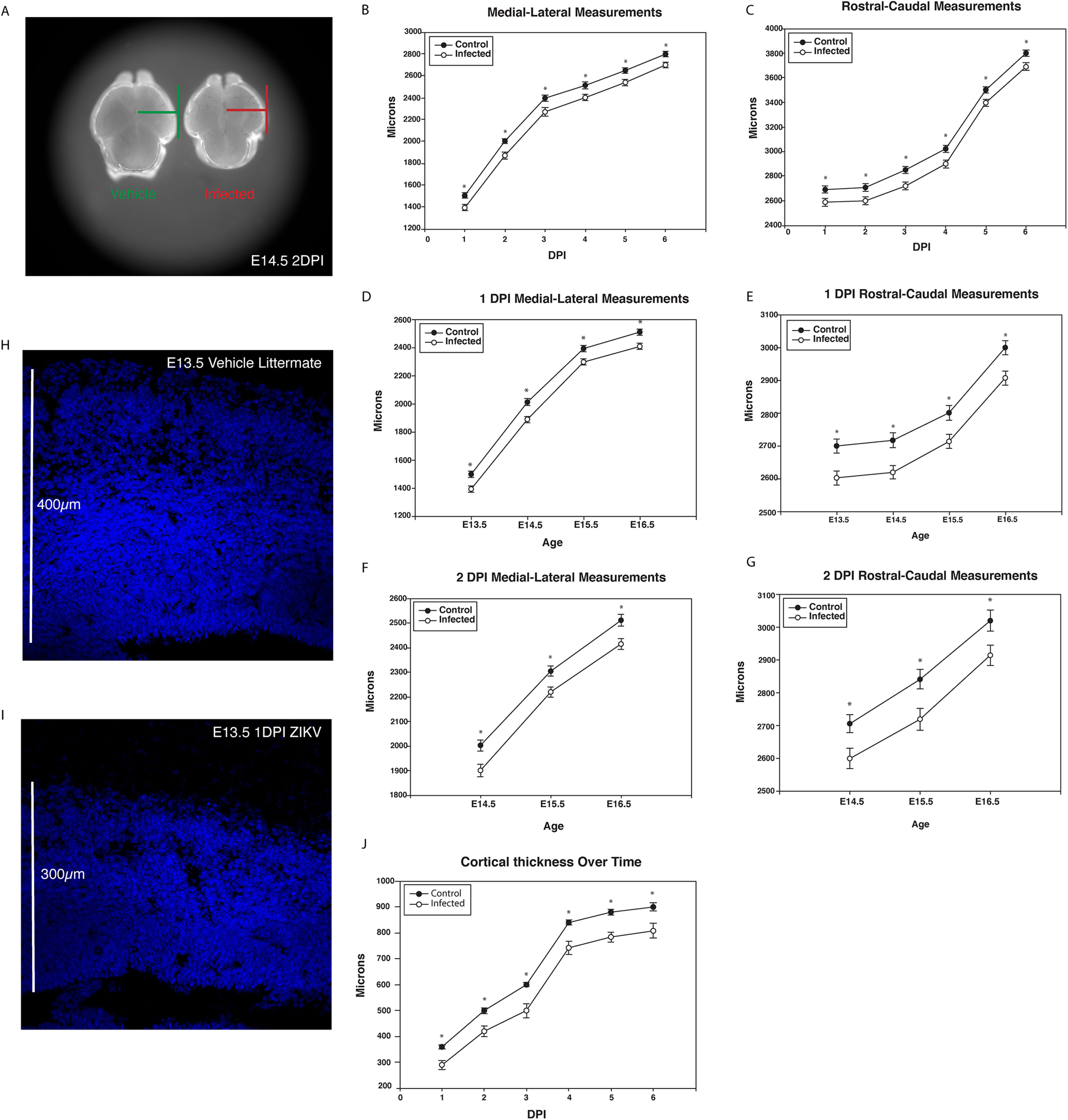
ZIKV decreased forebrain growth regardless of age of infection. Litters were injected at E12.5, E13.5, E14.5, and E15.5 and collected 24 h later. ***A***, Micrographs of example vehicle and ZIKV-infected brain from litter-matched embryos at E14.5 (2 DPI). Medial-lateral (***B***) and rostral-caudal (***C***) brain measurements were significantly decreased after ZIKV infection starting at 1 DPI. The microencephaly phenotype was not dependent on developmental age in (***D***) medial-lateral measurements at 1 DPI or (***E***) rostral-caudal measurements. Brains injected at E12.5, E13.5, and E14.5 and collected 48 h later also showed the same microencephaly phenotype in (***F***) medial-lateral and (***G***) rostral-caudal measurements. ***H***, Representative image of saline injected control neocortical wall collected at E13.5. ***I***, Image of ZIKV injected neocortical wall from the contralateral uterine horn collected at E13.5 (1 DPI). ***J***, Cortical thickness (micrometers from ventricle to pial surface) in 20× images of coronal brain sections. Control and infected brains were injected at E12.5 and collected 1–6 DPI. Statistical significance was calculated using unpaired *t* test (*p < 0.05) and error bars represent SE. An *n* of 4 was used for each condition.

To determine whether this mouse model of fetal ZIKV infection recapitulates the microencephaly phenotype found in humans and animal models, measurements of control and infected littermate brains were collected over time. In saline-infected or virus-infected littermates, we calculated brain size rather than head circumference to use a more reliable and repeatable method of measuring microencephaly. Control and infected brains were measured along the medial-lateral and rostral-caudal axes to assess the differences in forebrain size (Fig. 3*A*). When fetuses were injected at E12.5, microencephaly (>2 SDs below the mean) was found at all examined time points (1–6 DPI; Fig. 3*B*,*C*). In fact, viral infection on E12.5, 13.5, 14.5, or 15.5 generated microencephaly quickly, as early as 1 DPI. These time points span neurogenesis and infection times analogous to the second trimester of human gestation. Surprisingly, the ZIKV-induced decrease in brain size was not dependent on the age of infection when testing 1 and 2 DPI infection at E12.5, E13.5, E14.5, and E15.5 (Fig. 3*D–G*). Additionally, cortical thickness, measured at each time point after E12.5 infection, was consistently decreased from 1DPI to birth (example images of vehicle control in Fig. 3*H*, infected in Fig. 3*I*, and analysis in Fig. 3*J*). The ∼100-μm decrease in gross brain size was in line with the extent of reduction in cortical thickness, indicating that the decrease in brain size is primarily because of impaired cortical expansion (Fig. 3*H*,*I*).

**Figure 4.**
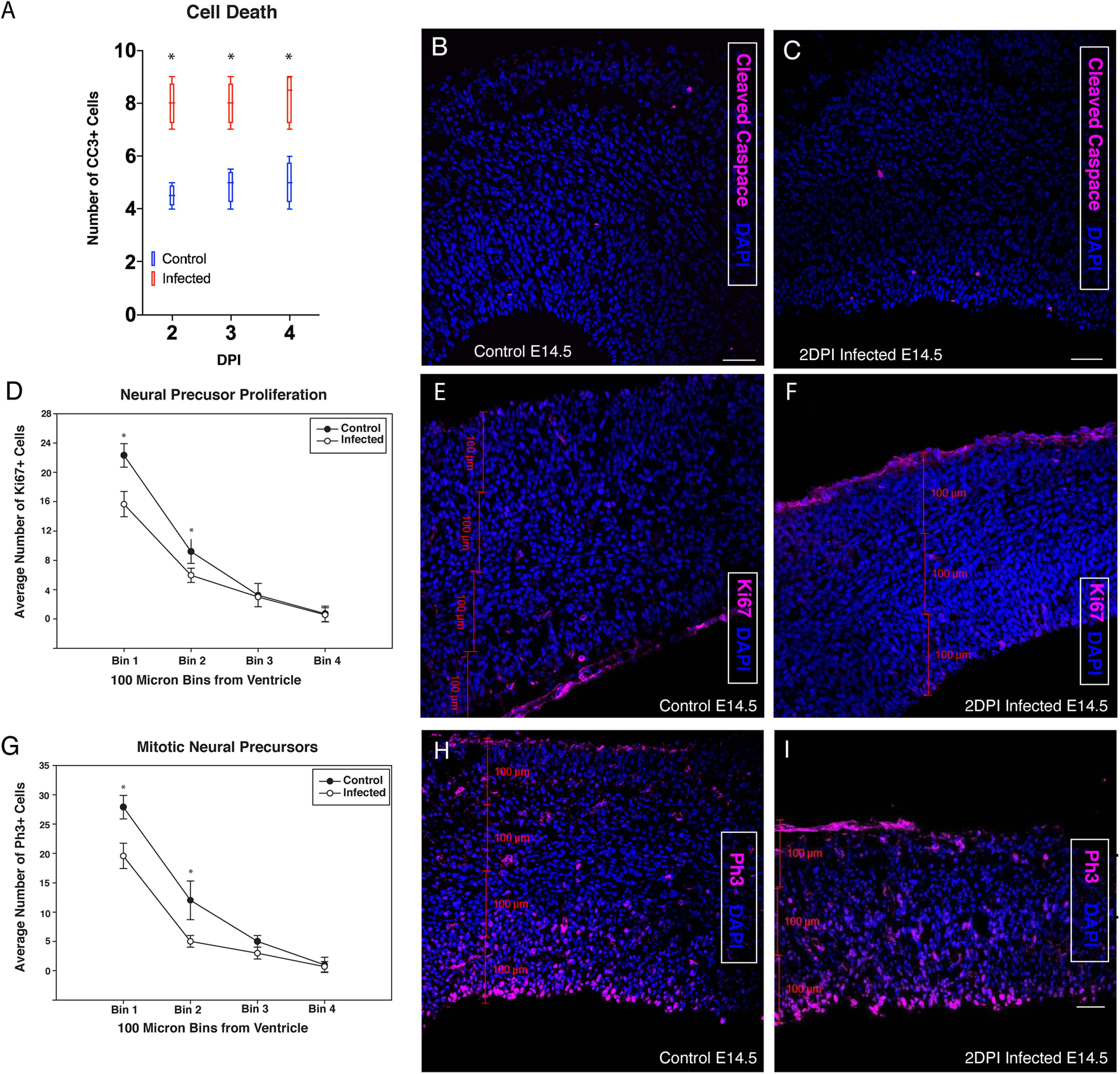
ZIKV infection increased cell death and reduced proliferation in the telencephalic germinal zones. ***A***, CC3^+^ cells counted in the neocortical wall of the future somatosensory cortex in vehicle (*n* = 4) and infected (*n* = 4) tissue in 2 DPI (E14.5), 3 DPI (E15.5), and 4 DPI (E16.5) tissue. Statistical significance was calculated using unpaired *t* test (*p < 0.05) and error bars represent SE. ***B***, E14.5 ZIKV-infected (2 DPI) cortical wall stained with CC3. ***C***, E14.5 vehicle control cortical wall stained with CC3. ***D***, Ki67+ average cell number counted in 100-μm bins (bin 1 closest to ventricle, bin 4 closest to pia) in embryos injected at E12.5 and collected at E14.5. Bins 1 and 2, which overlap most with the VZ and SVZ, have significant decreases in proliferating cells following ZIKV infection. ***E***, Representative single plane image of control on left and (***F***) infected on right. ***G***, PH3+ average cell number by bin. Bins 1 and 2, which overlap most with the VZ and SVZ, have significant decreases in proliferating cells following ZIKV infection**. *H***, Representative single plane image of control on left and (***I***) infected on right (*n* = 4, 4). Cell numbers were counted in each single plane image of 20-μm z-stack images and summed for each bin. Statistical significance was calculated using unpaired *t* test (*p < 0.05) and error bars represent SE.

To determine whether the observed decrease in cortical thickness was because of ZIKV-induced cell death, infected and control tissues were stained for CC3 to mark cells labeled for apoptosis. After 2 DPI of ZIKV exposure, there was a 29.3% increase in CC3^+^ apoptotic cells in the cortical wall, although the total number of apoptotic cells was still very low (Fig. 4*A–C*). Ki67 and PH3 were then used to assess changes in proliferation in infected and control brains. Decreased numbers of Ki67^+^ and PH3^+^ cells were found in the VZ of ZIKV-infected neocortex compared with saline-injected littermate controls (Fig. 4*D–I*). These findings indicate that the fetal ZIKV infection model resulted in increased cell death and a reduction in the number of proliferating NPCs during embryonic neural development. Both disruptions are likely to contribute to the observed microencephaly phenotype.

### Temporal infection of major precursor cell types

**Figure 5.**
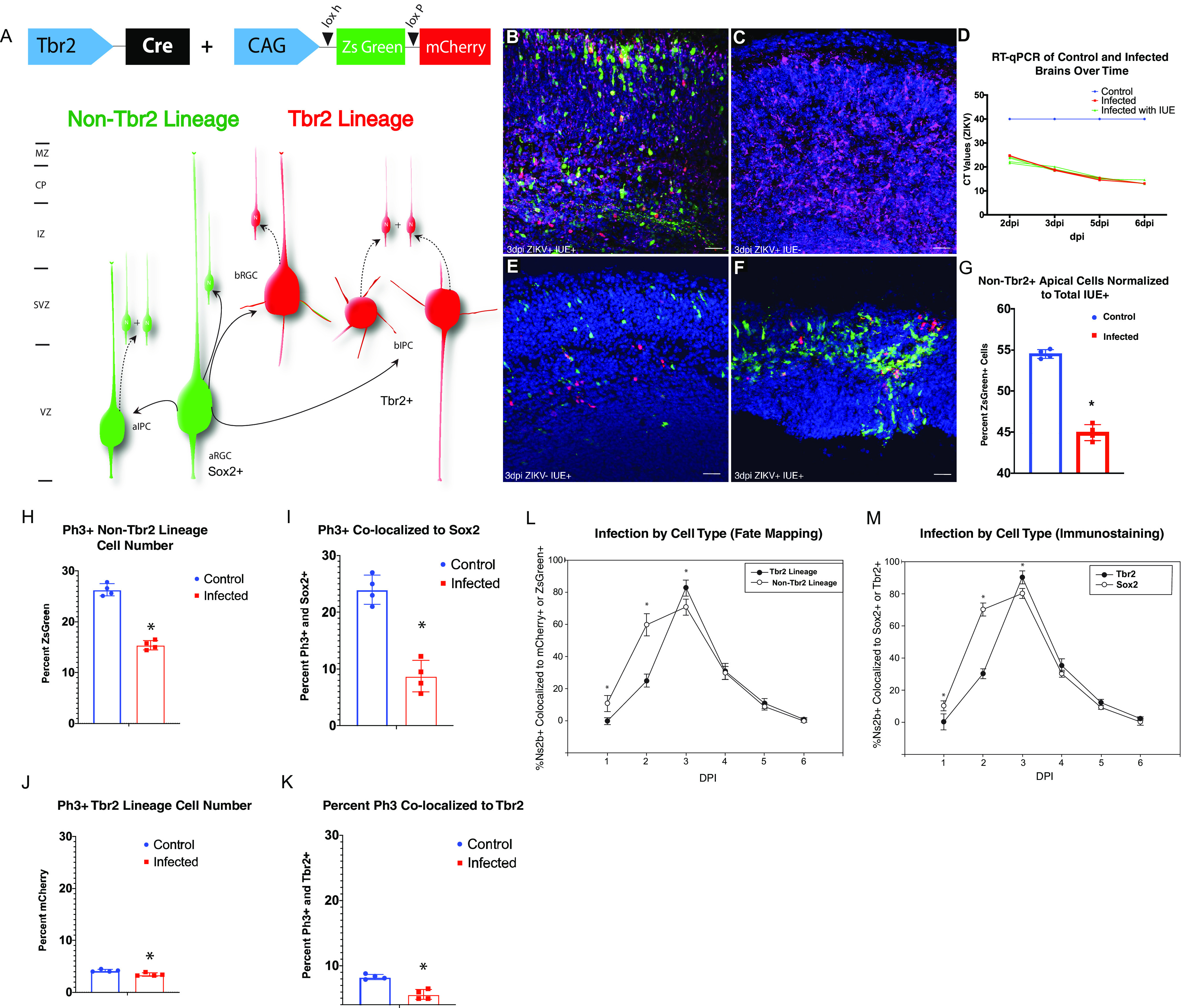
ZIKV causes a decrease in fate mapped apical precursor cell number. ***A***, Somatosensory cortex in control and infected brains was transfected with Tbr2-Cre + CAG-StopLight at E13.5 and collected 48 h later (E15.5), labeling the apical progenitors in green and the basal Tbr2 lineage progenitors in red. ***B***, 3 DPI (E15.5) infected tissue with and without (***C***) IUE show no difference in NS2B labeling. Scale bars: 50 μm. ***D***, ZIKV RT-qPCR in IUE and non-IUE-infected brains across time show no difference in infection. ***E***, Vehicle control electroporated with Tbr2-Cre and CAG-StopLight. Scale bar: 50 μm. ***F***, 3 DPI (E15.5) infected brain electroporated with Tbr2-Cre and CAG-StopLight. Scale bar: 50 μm. ***G***, The percentage of ZsGreen^+^ cells in the total electroporated population were reduced in infected tissue compared with control (*n* = 4, 4). Statistical significance was calculated using unpaired *t* test (*p < 0.05) and error bars represent SE. ***H***, Tbr2-Cre CAG-SL electroporated brains were collected after 48 h (IUE on E12.5). Control and infected brain sections were stained for PH3. The number of PH3^+^ Tbr2 lineage (mCherry^+^) cells was decreased by ZIKV infection. ***I***, The number of PH3^+^ non-Tbr2 lineage (ZsGreen^+^) cells were decreased by ZIKV infection. E14.5 control and infected brain sections were immunostained for PH3 and Tbr2. ***J***, The number of PH3^+^, Tbr2^+^ cells were decreased by ZIKV infection. ***K***, The number of PH3^+^, Sox2^+^ cells was substantially decreased by ZIKV infection. Statistical significance was calculated using unpaired *t* test (*p < 0.05) and error bars represent SE. A sample size of four was used in each condition. ***L***, Tbr2-StopLight fate mapping identifies temporal sequence of cell type infection. Brains were electroporated and co-infected with ZIKV at E12.5 and collected 1–6 DPI. Cell counts from confocal z-stacks indicate that ZsGreen^+^ apical precursors are preferentially infected at 1 and 2 DPI. At 3 DPI, more mCherry^+^ basal precursors are infected than apical precursors. Thereafter, infection decreased in both cell types (*n* = 4, 4). Statistical significance was calculated using unpaired *t* test (*p < 0.05) and error bars represent SE. ***M***, Immunohistochemical staining for apical (Sox2) and basal (Tbr2) precursors demonstrates temporal spread of infection through the germinal zones. Brains were injected with ZIKV at E12.5 and collected 1–6 DPI. Results indicate that Sox2^+^ apical precursors are preferentially infected at 1 and 2 DPI. At 3 DPI, more Tbr2^+^ basal precursors are infected. In later time points, both cell types are infected equally (*n* = 4, 4). Statistical significance was calculated using unpaired *t* test (*p < 0.05) and error bars represent SE.

To distinguish between apical and basal neocortical precursor cells, we used both immunophenotyping and genetic fate mapping approaches. *T-box brain protein 2* (Tbr2) was used as a cell lineage-specific promoter in combination with a Cre-recombinase-dependent dual color reporter. bIPCs were labeled with mCherry fluorescent protein, and non-Tbr2 lineage cells were labeled with ZsGreen fluorescent protein as previously shown by Tyler and Haydar (2013; Fig. 5*A*). In order to verify the results, we measured the compatibility of direct ZIKV infection with IUE labeling. We compared levels of viral infection and plasmid transfection between ZIKV, plasmid DNA, and a combination of the two. This assessed whether infectibility and the efficiency of these plasmid reporters were compromised by the combined approach. To account for variations in fetal development when comparing NPCs in infected and control mice, one uterine horn was injected with ZIKV/plasmid cocktail and the other was injected with plasmid cocktail and/or saline. IHC (Fig. 5*B–F*) and RT-qPCR (Fig. 5*D*) demonstrated no crossover of virus from the infected side to the control side or into the dam’s blood. The validity of this approach was confirmed as it resulted in the same plasmid transfection efficiency and ZIKV infection dynamics when ZIKV and IUE were conducted simultaneously. This allowed for the analysis of red and green cell numbers in control and infected tissue. At 3 DPI, infected tissue had significantly decreased numbers of ZsGreen^+^ cells, suggesting that the apical population of NPCs may be preferentially targeted by ZIKV (Fig. 5*G*).To assess whether ZIKV affected the proliferative capabilities of specific cell types, E12.5 embryos were infected with ZIKV and electroporated with Tbr2-Cre and CAG-StopLight before collection 48 h later. We found an 18.2% (Fig. 5*H*) decrease in mCherry/Ph3^+^ and a 40% decrease in the number of ZsGreen^+^/Ph3^+^ co-labeled cells in infected tissue compared with controls (Fig. 5*I*). To confirm that aRGCs were more affected than bIPCs at 2 DPI, non-electroporated ZIKV-infected brain samples were immunostained for Sox2 and Tbr2, marking aRGCs and bIPCs respectively. While both cell types showed decreased numbers of Ph3^+^ cells at 2 DPI, Sox2^+^ cells were particularly impacted with a 60% decrease compared with the 28.6% decrease found in Ph3^+^/Tbr2^+^ cells (Fig. 5*J*,*K*). These experiments confirmed that aRGCs are targeted by ZIKV early in infection and that proliferation of this essential NPC class is reduced.

#### Precursor type consequences of persistent ZIKV infection

Since aRGCs are particularly targeted in acute infection, we next sought to understand how longer infection times affect neocortical growth. Tbr2-Cre and CAG-StopLight electroporated tissue was stained with NS2B in control and infected tissue at 1 DPI through 6 DPI. ZsGreen^+^/NS2B^+^ and mCherry^+^/NS2B^+^ populations were counted on each day, and all cell counts were normalized to the total number of electroporated cells to account for the normal variability in the size of the electroporated field. Increased numbers of ZsGreen infected cells (ZsGreen^+^/NS2B^+^) were found at 1 and 2 DPI. However, at 3 DPI, the number of infected mCherry cells (mCherry^+^/NS2B^+^) surpassed the number of infected ZsGreen cells. The proportion of infected mCherry and ZsGreen cells equalized at 4–6 DPI as fewer NPCs were ZIKV^+^ over time (Fig. 5*L*,*M*). To further confirm cell type vulnerability, non-electroporated tissue was immunostained for Sox2 and Tbr2 at the same time points. Again, NS2B co-localized with more Sox2^+^ cells at 1 and 2 DPI. At 3 DPI, increased numbers of Tbr2^+^ mCherry cells co-localized with NS2B (Fig. 7*B*). These findings suggest that aRGCs are initially targeted by ZIKV and that aRGC-derived bIPCs gain active ZIKV infection either from cytoplasmic division or from virions shed from infected aRGCs.

#### ZIKV infection alters neocortical cytoarchitecture

**Figure 6.**
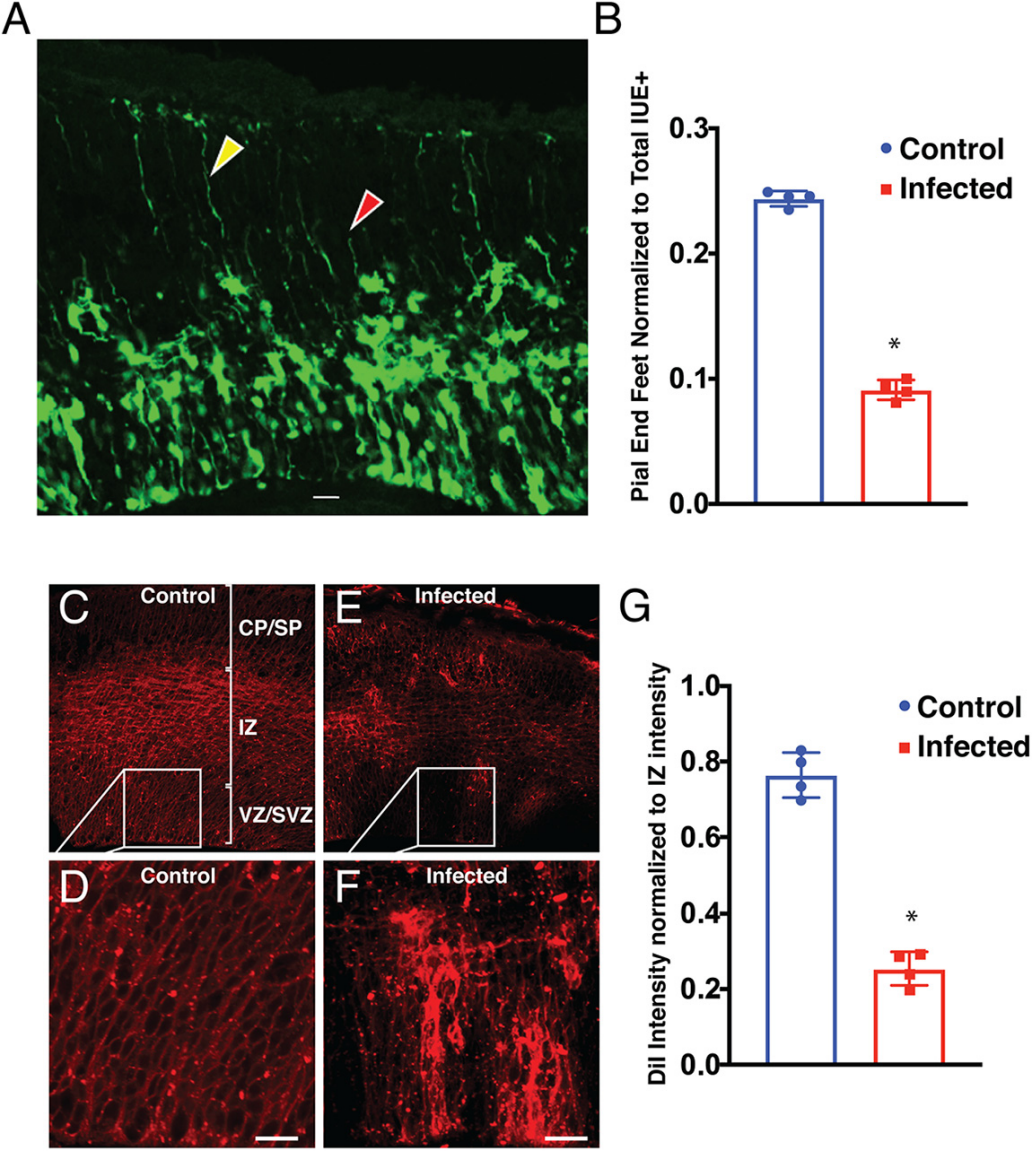
Forebrain ZIKV infection disrupts radial scaffolding of the telencephalic wall. Brains were electroporated with Tbr2-Cre + CAG-StopLight and either vehicle or ZIKV at E13.5 and collected 48 h later (E15.5). ***A***, ZIKV disrupts cytoarchitecture. Example of maximum projection z-stack of electroporated infected tissue at 2 DPI, showing normally developing (yellow arrow) and perturbed basal processes (red arrow). Scale bar: 20 μm. ***B***, Basal fiber end foot number is decreased in infected tissue. Cells were counted in the electroporated region of somatosensory cortex in 3D image stacks. The number of end feet was normalized to IUE^+^ cell number and was significantly decreased in infected tissue (*n* = 4, 4). Statistical significance was calculated using unpaired *t* test (*p < 0.05) and error bars represent SE. DiI was applied to the surface of E15.5, 2 DPI control and infected littermates and was allowed to diffuse for two weeks at room temperature. ***C***, DiI applied to the pial surface of the cortex labeled cell membranes in the VZ by diffusion down basal fibers. DiI labeling in the vehicle-injected cortex shows complete labeling of aRGC palisade from the pial to the ventricular surface. ***D***, Inset from ***C*** illustrates normal DiI intensity. Scale bar: 20 μm. ***E***, Basal fibers are disrupted in infected tissue. DiI labeling in 2 DPI-infected cortex reveals many areas of neocortical wall with patchy diffusion; somata and neurites are labeled in the IZ and CP but very little DiI label is found in the VZ and SVZ. ***F***, Inset from ***E*** illustrates sparse labeling of fibers in the VZ and SVZ of 2 DPI cortex. Scale bar: 20 μm. ***G***, DiI intensity in VZ/SVZ was normalized to the brightest intensity in the IZ directly above measurement taken in the VZ/SVZ. The VZ/SVZ intensity was significantly decreased in infected tissue (*n* = 4, 4). Statistical significance was calculated using unpaired *t* test (*p < 0.05) and error bars represent SE.

The labeling of aRGCs and bIPCs with the dual color fluorescent protein reporter allowed for visualization of changes in whole cell morphology in response to ZIKV infection. In particular, we found noticeable patterns of disorganization in the basal processes of aRGCs that stretch to the pial surface from the cell bodies within the VZ. aRGC basal fibers are required for neuronal migration during development and may elucidate the mechanism behind decreased neuronal migration after infection. Rosenfeld et al. (2017) described perturbed cytoarchitecture after ZIKV infection using organotypic mouse brain slice cultures electroporated with CAG-GFP but particular changes to the aRGC palisade have not yet been quantified. To test whether the number of basal fibers had decreased after infection, we counted the number of end feet reaching the pial surface in control and infected tissue at 2 DPI, normalized to the number of IUE^+^ cells present. While a decrease in aRGC fibers/endfeet may be because of the decrease in aRGC numbers after infection, the above fate mapping studies identified only an 18.2% decrease in apical ZsGreen^+^ cells at 2 DPI (Fig. 5*G*). In contrast, endfeet counts denote a 60% decrease in aRGC basal fibers at this same time point, indicating that the interrupted basal process phenotype is more pronounced than the decrease in numbers of aRGCs (Fig. 6*A*,*B*). Loss or retraction of aRGC basal fibers from the superficial cortical layers may lead to neuronal migration defects and ultimately cause decreased cortical thickness and hypoplasia.

To confirm the loss of aRGC basal processes, the lipophilic dye DiI was applied to the exterior surface of control and infected brains after the removal of the meninges. Allowing two weeks for passive intramembranous diffusion, we found that the density of DiI labeling was decreased in the VZ and SVZ of infected tissue. While neural projections and somata were DiI labeled in the cortical plate of infected tissue, the aRGC basal fibers were sparse in brains exposed to ZIKV compared with vehicle control brains (Fig. 6*C–G*). To quantify this difference, the DiI intensity was measured in the VZ and in the area of greatest fluorescence directly above the VZ in the intermediate zone (IZ). For each image, the VZ intensity was normalized to the IZ intensity to account for differences in DiI penetration between sections and brains. Infected tissue showed a ∼70% reduction in DiI intensity (Fig. 6*G*). Together, the IUE and DiI studies demonstrate that ZIKV infection causes disruption to the neocortical cytoarchitecture by disrupting aRGC basal processes.

### aRGC heterogeneity

In contrast to longstanding assumptions, recent data indicate that the aRGC population is heterogeneous and can undergo temporal transcriptomic change during the neurogenesis period (Miyata et al., 2004; Tyler et al., 2015; Heavner et al., 2020). In ZIKV-infected tissue, we found that a proportion of aRGC fibers were perturbed while others appeared to develop normally. This suggested that there may be intrinsic differences between these affected and non-affected cells that may stem from underlying aRGC heterogeneity. To test whether subpopulations of aRGCs may be differentially susceptible to infection by ZIKV, we took advantage of a recent scRNAseq study in the E15.5 mouse neocortex (Li et al., 2020). This dataset reveals two distinct clusters of cortical aRGCs (aRGC1 and aRGC2). We sought to determine whether genes differentially expressed between these two groups may identify separate populations of aRGCs with differential vulnerability to ZIKV infection.

**Figure 7.**
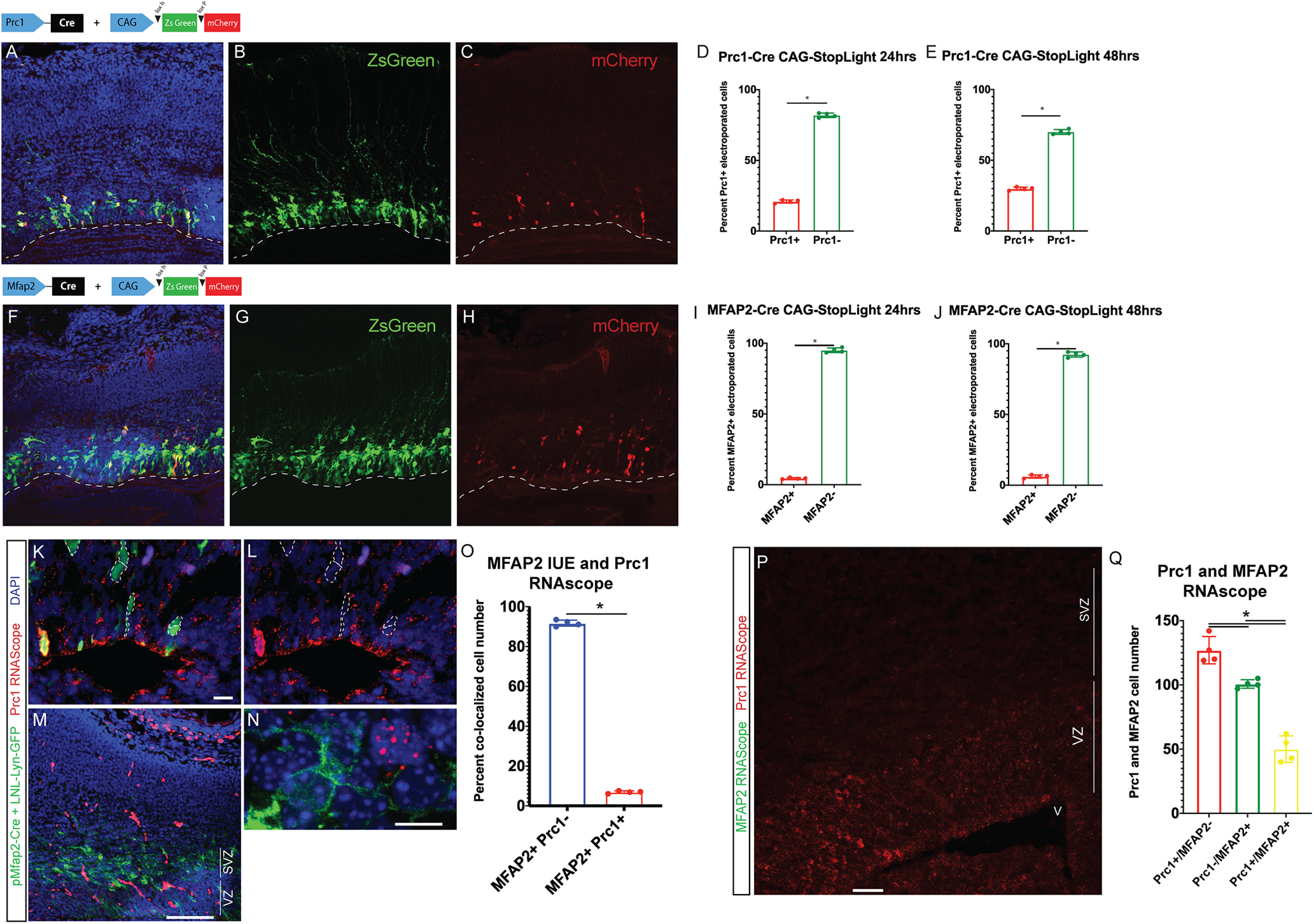
Fate mapping of aRGC types. E14.5 mice were electroporated with *Prc1*-Cre or *MFAP2*-Cre and CAG-StopLight and collected 24 or 48 h later. ***A***, IUE with *Prc1*-Cre and CAG-StopLight produced VZ-localized mCherry^+^ cells. A composite image from *Prc1*-Cre experiments with non-specific electroporated cells in green (***B***) and (***C***) *Prc1*^+^ cells in red. Scale bar: 50 μm. Red and green cell number were counted and normalized to the total number of electroporated cells for (***D***) 24-h and (***E***) 48-h time points. ***F***, IUE with *MFAP2*-Cre and CAG-StopLight produced VZ localized mCherry^+^ cells. A composite image from *MFAP2*-Cre and CAG-SL recombination allowing visualization of electroporated cells in green (***G***) and (***H***) *MFAP2*^+^ cells in red. Scale bar: 50 μm. Red and green cell number were counted and normalized to the total number of electroporated cells for 24-h (***I***) and 48-h (***J***) time points (*n* = 4 per condition). Statistical significance was calculated using unpaired *t* test (*p < 0.05) and error bars represent SE. ***K***, E13.5 mice were electroporated with *MFAP2*-Cre and CAG-LNL-LYN-GFP and collected 48 h later. *Prc1*^+^ cells were labeled with RNAscope (red), electroporated *MFAP2*+ cells with GFP and nuclei were labeled with Hoechst (blue). Scale bar: 15 μm. ***L***, *Prc1* RNAscope alone. ***M***, 20× magnification image with no zoom shows marker labeling in the VZ and SVZ 48 h after electroporation. Scale bar: 100 μm. ***N***, Zoom of a *Prc1*^+^ cell in red neighboring a *MFAP2*^+^ in green. Scale bar: 10 μm. ***O***, Cells counted positive for RNAscope by including two or more puncta within the cell body that were co-localized with GFP from electroporation were normalized to the total number of electroporated cells in the field to determine percent co-localized cell number. Seven percent of the electroporated cells were positive for *Prc1* RNAscope (*n* = 4). Statistical significance was calculated using unpaired *t* test (*p < 0.05) and error bars represent SE. ***P***, E15.5 cryosectioned tissue was stained for *MFAP2* (green), and *Prc1* (red) using RNAscope. Scale bar: 50 μm. ***Q***, *Prc1*^+^ cells, *MFAP2*^+^ cells, and co-labeled cells were counted in 20-μm z-stack images (*n* = 4). Statistical significance was calculated using a one-way ANOVA (*p < 0.05) and error bars represent SE.

To develop markers for these two clusters of aRGCs, differential gene expression in aRGC1 and aRGC2, compared with gene expression profiles of other NPC and neuron clusters, was used to create a list of potential transcriptional markers specific to aRGC1 cells or to aRGC2 cells. *Prc1* and *AXL* were top candidate genes for aRGC1 and *MFAP2* and *Robo4* were top candidate genes for aRGC2 (Table 1). Conserved regulatory regions proximal to the transcriptional start sites of these candidate genes were used to construct Cre-driver plasmids, which were tested by IUE using the CAG-StopLight reporter plasmid at 24 and 48 h. This approach yielded mCherry^+^ recombinant cells in the VZ with the expected morphology and location of aRGCs (Fig. 7*A–C*). *Prc1*-Cre labeled a larger proportion of cells compared with *MFAP2*-Cre (7D-E). *Prc1*^+^ cells also showed greater proliferative potential compared with *MFAP2*^+^ cells as seen in the proportion of mCherry^+^ cells counted between 24 and 48 h (Fig. 7*I*,*J*).

**Table 1:** Group 1 and group 2 aRGC markers chosen from scRNAseq data

Group	Fold change	*p* value
Group 1, Prc1	6.3	5.60E-35
Group 1, Axl	2.3	9.87E-03
Group 2, MFAP2	0.64	1.54E-08
Group 2, Robo4	6.2	1.30E-32

The fold change represents differential expression between aRGC1 and aRGC2. For details, see Materials and Methods.

**Table 2:** Key resource table

Key resources table				
Reagent type or resource	Designation	Source or reference	Identifiers	Additional information
Sequence-based reagent	Prc1 ISH probe	Advanced Cell Diagnostics	57712	MFISH
Sequence-based reagent	AXL ISH probe	Advanced Cell Diagnostics	450931	MFISH
Sequence-based reagent	MFAP2 ISH probe	Advanced Cell Diagnostics	445421	MFISH
Sequence-based reagent	Robo4 ISH probe	Advanced Cell Diagnostics	466321	MFISH
Sequence-based reagent	V-ZIKA-06 ISH probe	Advanced Cell Diagnostics	5\ill\5\ill\1	MFISH
Antibody	Ns2b	Genetex	GTX133308	Anti-rabbit (1:250)
Antibody	CC3	Cell Signaling	9661-s	Anti-rabbit (1:250)
Antibody	Ki67	BS Pharmigen	MAB1567	Anti-mouse (1:250)
Antibody	Ph3	Millipore	06-570	Anti-rabbit (1:300)
Antibody	Tbr2	Millipore, Santa Cruz	AB2283, sc-293481	Anti-rabbit (1:300), Anti-mouse (1:250)
Antibody	Sox2	Santa Cruz	sc-17320	Anti-goat (1:250)
Antibody	Tuj1	Santa Cruz	sc-\?\0005	Anti-mouse (1:250)
Secondary	Goat anti-rabbit	Invitrogen	A11008	Goat anti-rabbit (1:500)
Secondary	Goat anti-mouse	Invitrogen	A21050	Goat anti-mouse (1:500)
Secondary	Donkey anti-mouse	Invitrogen	A2202	Donkey anti-mouse (1:500)
Secondary	Donkey anti-rabbit	Invitrogen	A31573	Donkey anti-rabbit (1:500)
Vector	Tbr2 Cre	Tyler et al. (2015)	IUE	
Vector	Prc1 Cre	Available upon request	IUE	
Vector	MFAP2 Cre	Available upon request	IUE	
Vector	CAG-StopLight	Montana Molecular	IUE	
Vector	CAG-LNL-LYN-GFP	Available upon request	IUE	
Software, algorithm	R	https://www.r-project.org	SCR 001905	
Software, algorithm	Seurat	https://\ill\.org/seurat/	RRID:SCR_007322	
Software, algorithm	Fiji	https://imagej.net/Fiji	RRID:SCR_002285	
Software, algorithm	Prism	https://www.graphpad.com/scientific-software/prism/	RRID:SCR_002798	
Software, algorithm	Sigma Plot	https://systatsoftware.com/downloads/download-sigmaplot/		
Primers	Prc1 forward	TACCGTTCTCCGTCCCGCTCGAGGGGCAGAGCCG		
Primers	Prc1 reverse	GCTCTGCCCCTCGAGCGGGACGGAGAACGG		
Primers	Prc1 forward	GGCGAATTGGGTACCCTTGGCTTGCTAGGGTGTGA		
Primers	Prc1 reverse	CCCTAGCAAGCCAAGCGCTATC		
Primers	Robo4 forward	GGCGAATTGGGTACCCATGCATTTGGAGTTTCCATGTCCT		
Primers	Robo4 reverse	GCTCTGCCCCTCGAGGGCTGCTCTCGGCTCC		
Primers	MFAP2 forward	GGCGAATTGGGTACCACTCGATCTCCCTTAATCTGCCT		
Primers	MFAP2 reverse	GGCGAATTGGGTACCACTCGATCTCCCTTAATCTGCCT		
Primers	ZIKV forward	AARTACACATACCARAACAAAGTGGT		
Primers	ZIKV reverse	TCCRCTCCCYCTYTGGTCTTG		

**Table 3:** Statistics table

Figure	Experiment	*n*	Data structure	Type of test	*p* value	SE control	SE infected	SE
3*B*	1 DPI ML	4	Normal distribution	*t* test	0.00684739	0.00	0.00	
3*C*	1 DPI RC	4	Normal distribution	*t* test	0.000128	0.00	0.00	
3*B*	2 DPI ML	4	Normal distribution	*t* test	0.00136	0.00	0.00	
3*C*	2 DPI RC	4	Normal distribution	*t* test	0.00349	0.00	0.01	
3*B*	3 DPI ML	4	Normal distribution	*t* test	0.00017204	0.00	0.01	
3*C*	3 DPI RC	4	Normal distribution	*t* test	0.0688023	0.01	0.01	
3*B*	4 DPI ML	4	Normal distribution	*t* test	0.0146552	0.01	0.02	
3*C*	4 DPI RC	4	Normal distribution	*t* test	0.0209259	0.01	0.033	
3*B*	5 DPI ML	4	Normal distribution	*t* test	0.01277896	0.04	0.03	
3*C*	5 DPI RC	4	Normal distribution	*t* test	0.03849331	0.59	0.49	
3*B*	6 DPI ML	4	Normal distribution	*t* test	0.000118	0.01	0.01	
3*C*	6 DPI RC	4	Normal distribution	*t* test	0.00028544	0.02	0.01	
3*D*	1 DPI 1 ML	4	Normal distribution	*t* test	0.00684739	0.00	0.00	
3*E*	1 DPI 1 RC	4	Normal distribution	*t* test	0.000128	0.00	0.00	
3*D*	1 DPI 2 ML	4	Normal distribution	*t* test	0.0013651	0.01	0.01	
3*E*	1 DPI 2 RC	4	Normal distribution	*t* test	0.00349342	0.01	0.01	
3*D*	1 DPI 3 ML	4	Normal distribution	*t* test	0.00478629	0.01	0.01	
3*E*	1 DPI 3 RC	4	Normal distribution	*t* test	0.00169226	0.01	0.00	
3*D*	1 DPI 4 ML	4	Normal distribution	*t* test	0.00010226	0.01	0.01	
3*E*	1 DPI 4 RC	4	Normal distribution	*t* test	0.00169226	0.01	0.00	
3*F*	2 DPI 1 ML	4	Normal distribution	*t* test	0.00136	0.01	0.01	
3*G*	2 DPI 1 RC	4	Normal distribution	*t* test	0.00349	0.00	0.01	
3*F*	2 DPI 2 ML	4	Normal distribution	*t* test	0.00495933	0.01	0.01	
3*G*	2 DPI 2 RC	4	Normal distribution	*t* test	0.001435	0.01	0.00	
3*F*	2 DPI 3 ML	4	Normal distribution	*t* test	0.00695174	0.01	0.01	
3*G*	2 DPI 3 RC	4	Normal distribution	*t* test	0.00914545	0.01	0.01	
3*J*	1 DPI	4	Normal distribution	*t* test	0.00180085	9.49	3.19	
3*J*	2 DPI	4	Normal distribution	*t* test	2.7878E-07	0.64	3.83	
3*J*	3 DPI	4	Normal distribution	*t* test	3.3158E-07	3.11	2.67	
3*J*	4 DPI	4	Normal distribution	*t* test	3.4267E-06	5.12	3.79	
3*J*	5 DPI	4	Normal distribution	*t* test	3.3551E-05	6.57	5.81	
3*J*	6 DPI	4	Normal distribution	*t* test	3.865E-06	6.14	2.29	
4*A*	2 DPI	4	Normal distribution	*t* test	0.0002572	0.20	0.41	
4*A*	3 DPI	4	Normal distribution	*t* test	0.00091272	0.31	0.41	
4*A*	4 DPI	4	Normal distribution	*t* test	0.00208317	0.41	0.48	
4*D*	Bin 1	4	Normal distribution	*t* test	0.0384043	2.17	0.48	
4*D*	Bin 2	4	Normal distribution	*t* test	0.02149562	0.50	2.21	
4*D*	Bin 3	4	Normal distribution	*t* test	0.8053496	2.51	1.60	
4*D*	Bin 4	4	Normal distribution	*t* test	0.74358669	0.25	0.00	
4*G*	Bin 1	4	Normal distribution	*t* test	0.00111198	1.31	1.55	
4*G*	Bin 2	4	Normal distribution	*t* test	0.02065501	1.04	1.19	
4*G*	Bin 3	4	Normal distribution	*t* test	0.07577485	1.29	0.85	
4*G*	Bin 4	4	Normal distribution	*t* test	0.1339746	1.11	0.00	
5*G*	Non-Tbr2	4	Normal distribution	*t* test	2.454E-06	0.68	1.17	
5*H*	Ph3 Tbr2	4	Normal distribution	*t* test	0.01009862	0.01	0.00	
5*I*	Ph3 non Tbr2	4	Normal distribution	*t* test	2.6496E-06	0.01	0.01	
5*J*	Ph3 Tbr2	4	Normal distribution	*t* test	0.00071178	0.15	1.04	
5*K*	Ph3 sox2	4	Normal distribution	*t* test	0.00016526	0.83	0.38	
5*L*	1 DPI	4	Normal distribution	*t* test	7.7206E-06	1.26	0.38	
5*L*	2 DPI	4	Normal distribution	*t* test	1.4818E-07	3.21	1.46	
5*L*	3 DPI	4	Normal distribution	*t* test	0.00011085	2.25	1.70	
5*L*	4 DPI	4	Normal distribution	*t* test	0.59430986	1.18	1.59	
5*L*	5 DPI	4	Normal distribution	*t* test	0.90448279	1.43	1.05	
5*L*	6 DPI	4	Normal distribution	*t* test	0.27823572	0.31	0.38	
5*M*	1 DPI	4	Normal distribution	*t* test	6.4046E-06	0.78	0.25	
5*M*	2 DPI	4	Normal distribution	*t* test	0.00029411	1.78	1.29	
5*M*	3 DPI	4	Normal distribution	*t* test	0.00011655	1.25	2.24	
5*M*	4 DPI	4	Normal distribution	*t* test	0.32147813	1.66	1.10	
5*M*	5 DPI	4	Normal distribution	*t* test	0.24536482	1.10	1.21	
5*M*	6 DPI	4	Normal distribution	*t* test	0.53696332	0.12	0.06	
6*B*	endfeet	4	Normal distribution	*t* test	0.03248771	0.03	0.02	
6*G*	DiI	4	Normal distribution	*t* test	0.00082241	0.04	0.05	
7*D*	Prc1 24 h	4	Normal distribution	*t* test	1.5942E-10	1.29	1.64	
7*E*	Prc1 48 h	4	Normal distribution	*t* test	2.7202E-12	0.97	1.24	
7*I*	MFAP2 24 h	4	Normal distribution	*t* test	8.9982E-11	0.69	0.95	
7*J*	MFAP2 48 h	4	Normal distribution	*t* test	2.2807E-10	0.10	0.95	
7*O*	IUE and RNAscope	4	Normal distribution	*t* test	8.0791E-11	0.10	0.98	
7*Q*	Double RNAscope	4	Normal distribution	ANOVA	0.0001	1.08	1.81	1.05
8*B*	MFAP2 Prc1 infected	4	Normal distribution	*t* test	1.3469E-05	1.37	0.88	
8*D*	Prc1 AXL	4	Normal distribution	*t* test	9.249E-10	1.45	0.56	
8*F*	MFAP2 AXL	4	Normal distribution	*t* test	1.4042E-08	1.11	0.91	
8*H*	Prc1 AXL ZIKV	4	Normal distribution	*t* test	4.8802E-12	0.92	0.27	

The candidate marker genes for each cortical aRGC type were also used as targets for multiplexed single molecule *in situ* hybridization. *Prc1* RNAscope hybridization was performed on 24 h *MFAP2-Cre* + CAG-LNL-Lyn-GFP electroporated tissue to determine whether these markers indeed label different aRGCs. It was found that only 7% of the *MFAP2*^+^ electroporated cells were also positive for *Prc1* transcript (Fig. 7*K–O*). Using multiplexed hybridization of *Prc1* and *MFAP2* RNAscope probes recapitulated results showing that there is separation of marker expression in these cell types and that there are more *Prc1+* only cells followed by *MFAP2^+^* only cells, with the smallest proportion being *Prc1^+^/MFAP2*^+^ cells (Fig. 7*P*,*Q*).

**Figure 8.**
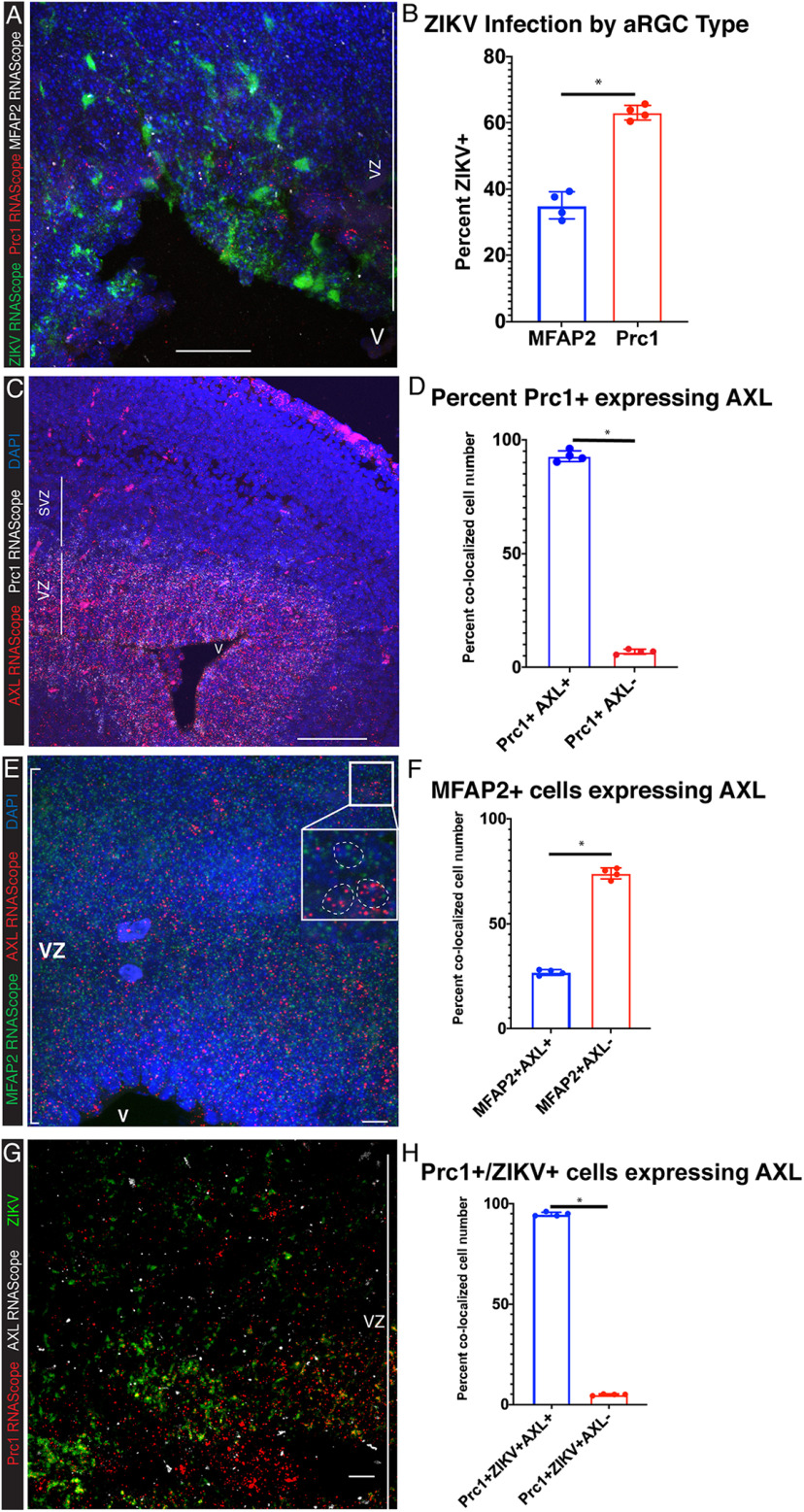
*MFAP2*^+^ aRGCs show lower infection rates compared with *Prc1*^+^ aRGCs. ***A***, Multiplexed RNAscope staining shows fewer infected *MFAP2*^+^ cells compared with *Prc1*^+^ cells. E15.5 2 DPI infected tissue was stained for ZIKV (green), *Prc1* (red), and *MFAP2* (white) using RNAscope. Scale bar: 50 μm. ***B***, *Prc1*^+^ cells are more vulnerable to ZIKV infection than *MFAP2*^+^ cells. Statistical significance was calculated using unpaired *t* test (*p < 0.05) and error bars represent SE (*n* = 4). ***C***, *Prc1* and *AXL* are highly expressed in the telencephalic germinal zone. E15.5 tissue was stained for *AXL* (red) and *Prc1* (white) using RNAscope. Scale bar: 80 μm. ***D***, Over 90% of *Prc1*+ cells co-express *AXL* (*n* = 4). Statistical significance was calculated using unpaired *t* test (*p < 0.05) and error bars represent SE. ***E***, The majority of *MFAP2*^+^ cells are negative for *AXL* expression. The white box outlines a zoomed example of *MFAP2*^+^ cells with and without *AXL* co-expression showing variability of *AXL* expression in *MFAP2*^+^ cells. Scale bar: 10 μm. ***F***, *AXL* expression is decreased in *MFAP2*^+^ cells compared with *Prc1*^+^ cells. Despite the lower infection rate in group 2 aRGCs, 26.5% of *MFAP2*^+^ cells co-expressed *AXL*. ***G***, ZIKV infection correlates with AXL expression in *Prc1*^+^ cells. E15.5 tissue was stained for *Prc1* (red) and *AXL* (white) and ZIKV (green) using RNAscope. Scale bar: 10 μm**. *H***, The majority of *Prc1*^+^, ZIKV^+^ cells also express *AXL*. Over 95% of infected *Prc1*^+^ cells were co-labeled with *AXL* RNAscope. Statistical significance was calculated using unpaired *t* test (*p < 0.05) and error bars represent SE (*n* = 4).

Given the separation of *Prc1*^+^ and *MFAP2*^+^ populations, we next tested whether these groups were differentially vulnerable to ZIKV infection by multiplexing RNAscope probes against *Prc1*, *MFAP2*, and ZIKV. ZIKV was found to be co-labeled in *Prc1*^+^ cells significantly more often than in *MFAP2*^+^ cells. In fact, at 2 DPI, 63% of *Prc1*^+^ cells were ZIKV-infected while only 35% of *MFAP2*^+^ cells were infected (Fig. 8*A*,*B*).

### AXL as a group 1 identifier and candidate viral entry receptor

Despite evidence that *AXL* is sufficient but not necessary to enable ZIKV entry (Wells et al., 2016), we sought to test whether aRGC1 vulnerability to ZIKV was correlated with *AXL* expression. RNAscope of *Prc1 *+* AXL* and *MFAP2* + *AXL* corroborated the scRNAseq data and showed that *AXL* mRNA levels are higher in aRGC1/*Prc*1^+^ cells compared with aRGC2/*MFAP2*^+^ cells (Fig. 8*C*,*D*). However, 26% of *MFAP2*^+^ cells did express *AXL*, showing that *AXL* expression is not exclusive to aRGC1 (Fig. 8*E*,*F*). When multiplexing *AXL* with *ZIKV* and the respective group marker, we found that *AXL*, *Prc1*, and ZIKV were co-expressed at high levels (Fig. 8*G*,*H*). This correlation between *AXL* expression and ZIKV infection is consistent with previous findings *in vitro* (Miner and Diamond, 2016; Nowakowski et al., 2016; Meertens et al., 2017). These findings do not explain why no significant difference has been found between AXL-deficient mice and their littermates reported in Wang et al. (2017), which implicate viral entry receptors other than AXL for ZIKV infection. Future studies should leverage scRNAseq data to mark multiple viral entry receptors with RNAscope to further understand how *AXL* interacts with other entry receptors in aRGC ZIKV infection.

## Discussion

Determining the mechanisms by which ZIKV alters fetal brain growth and function can enable more focused therapies to support the lives of children with congenital Zika syndrome. Details about the route of infection, the cell types most impacted by the virus, and the temporal change in proliferative and postmitotic zones of the growing brain are needed to provide the best framework for future supportive care for those exposed to ZIKV during fetal life. We therefore developed an *in vivo* model of ZIKV brain infection, using direct forebrain intraventricular injection, that enables this type of longitudinal, cell type-specific analysis without the need for immune system attenuation or virus inactivation. This approach of infecting the mouse brain at different timepoints in pregnancy and then collecting infected and control embryos 1–6 DPI allowed for a measurement of the progression of ZIKV infection.

The concentration and strain of virus used here uncovered less cell death and more morphologic differences in the developing cells in the forebrain. We found that ZIKV passes first through a subpopulation of aRGCs expressing high levels of *AXL*, then transits to intermediate progenitors. Furthermore, while the viral infection causes devastating loss to certain cell groups, other precursors and postmitotic neurons appear less affected. Even at the latest time points examined, key structural features of the neocortex are maintained, and many neurons survive despite harboring viral infection.

The ability to assess infection across time enabled us to determine whether the severity of outcome depends on the age at which infection first occurs. Surprisingly, the severity of microencephaly was not dependent on the developmental stage during infection. The consistent microencephaly observed across developmental stages may be a consequence of the under-developed mouse immune system, as the first immune response to antigens is not found until the first postnatal week (Holsapple et al., 2003). Measurements of cell death and proliferation showed that viral infection increases the number of cells marked for apoptosis while decreasing the number of proliferating cells, both of which can result in microencephaly. The disruption in aRGC basal fibers likely contributes to the microencephaly phenotype as well by interrupting this key substrate of neuronal migration. This interference in migration may also play a role in the cell death seen postnatally (Dudley et al., 2017).

Our model allowed for *in vivo* time course studies to measure the progression of ZIKV infection over the course of 6 d of prenatal brain development. This is the longest *in vivo* time course study of a ZIKV model to date. This allowed for tracking of the microencephaly phenotype over time and elucidated some surprising results. We found that severity of microencephaly in this model was consistent across DPI1–DPI6. One explanation for this is that certain aRGCs are targeted initially, creating a severe depletion in their proliferation and neurogenesis which is then compensated for by the remaining aRGCs and other NPC types. Alternatively, as soon as new aRGCs are born and able to proliferate, cortical expansion continues at the same rate, but is overall unable to compensate for the initial volume loss.

It was also surprising that the microencephaly phenotype was not impacted by the age of the animal at the start of infection given that human data shows increased severity of infections occurring earlier in pregnancy (Johansson et al., 2016; Schuler-Faccini et al., 2016). This is likely because of the differences in human and murine immune development, to species-specific differences in plasticity of the germinal zones, or to the condensed timeframe of cortical development in mouse compared with human. Unlike humans, mice do not have fully developed immune systems until one week after birth. Thus, there may be additional inflammatory effects on human brain following ZIKV infection compared with mouse. The E13.5–E16.5 time frame covers the peak of mouse neurogenesis. Some differences would have been expected between E13.5 and E16.5, however, it may be possible that the difference in aRGC number is not great enough in this narrow window to see differences. If it were more technically possible, starting the IUE timepoints even earlier would have helped further explain the progression of ZIKV infection in causing microencephaly. Additionally, a proliferation compensation mechanism may be in play. This is an interesting avenue for future study given the role that cell-to-cell contact plays in proliferation. If cell density in the germinal zones is decreased because of ZIKV-induced cell death, the decreased pressure on cell contact could allow for transiently increased proliferation in the unaffected cell populations. While ours and other data suggest decreased proliferation overall, longer time courses of proliferation still need to be tested.

The cell type vulnerability identified in this study identifies precursor groups that differ in their susceptibility to the virus. For example, we found greater defects in proliferation and morphologic development in apical precursor cells, which are likely responsible for the rapid expression of microencephaly within a day after infection. While aRGCs are infected preferentially at 1–2 DPI, bIPCs were the largest infected population at 3 DPI. This may explain the discrepancy in previous findings of aRGCs being the primary target versus others who found IPCs to be ZIKV’s primary target (Onorati et al., 2016; Lin et al., 2017). However, as microencephaly did not significantly worsen over time after infection in the mouse, the initial and substantial impact on proliferation appears to be compensated by continued proliferation in the remaining aRGCs and IPCs. Fate mapping with the IUE-based Cre/LoxP recombination system identified that diversity in the aRGC population may underlie growth dynamics in brains infected with ZIKV. In particular, we provide evidence that *MFAP2*^+^ aRGCs are less susceptible to ZIKV infection, indicating that this group of cells, and their progeny, may underlie the compensatory proliferation described above. One possible mechanism responsible for the differential vulnerability between *Prc1*^+^ cells and *MFAP2*^+^ cells is the increased expression of *AXL* in the *Prc1*+ cells. However, these findings also suggest that *AXL* is not necessary for infection, as 4% of ZIKV^+^ cells were *AXL*^–^. The presented data corroborate the findings of Nowakowski et al. (2016) who showed that *AXL* is highly expressed in aRGCs and functions as a viral entry receptor; these data are also supportive of the results of Wells et al. (2016), which demonstrated that cells could become infected without the presence of *AXL* (Onorati et al., 2016; Wells et al., 2016). One *AXL*-independent means of viral infection could be the passage of viral RNA during cell division. Although these results as well as previous research indicate that mitosis is impaired during infection (Onorati et al., 2016; Tang et al., 2016; Dudley et al., 2017; Lin et al., 2017; Rosenfeld et al., 2017), if an already infected aRGC is able to successfully divide, it could pass infection to its bIPC daughter cell. Nevertheless, the high correlation between *Prc1* expression, *AXL* expression, and ZIKV infection make *AXL* an interesting group 1 candidate marker gene. This model provides a framework for additional candidate viral entry research aimed at designing chemical inhibitors. It also suggests that interference of AXL-mediated endocytosis will decrease infection rates in the highly targeted aRGC1 population, potentially avoiding the severe and early onset of microcephaly as indicated by previous work showing decreased ZIKV infection from decreased *AXL* expression (Wells et al., 2016).

These results not only increase understanding of how ZIKV infection impacts neural development, but also indicate that a subtype of aRGCs, which previously were believed to be a largely homogenous population, may be critically involved in the initiation of infection. Li et al. (2020) showed transcriptional priming and pseudotime evidence that aRGC1 (*Prc1*^+^) may be fated toward direct neurogenesis while aRGC2 (*MFAP2*^+^) cells are primed toward the Tbr2^+^ bIPC lineage (Li et al., 2020). Similarly, another study recently described aRGC heterogeneity based on Sox9 expression (Fabra-Beser et al., 2020), which is also differentially expressed between aRGC1 and aRGC2 groups (log fold change: −0.62, adjusted *p* value: 0.00022). Future work using tools designed to study cortical circuits subserved by aRGC1 and aRGC2 progeny will highlight specific changes in neural architecture likely disturbed in people with congenital Zika syndrome.

## Synthesis

Reviewing Editor: Susanne Wolf, Charite University Hospital Decisions are customarily a result of the Reviewing Editor and the peer reviewers coming together and discussing their recommendations until a consensus is reached. When revisions are invited, a fact-based synthesis statement explaining their decision and outlining what is needed to prepare a revision will be listed below. The following reviewer(s) agreed to reveal their identity: Zoltan Simandi.

Both reviewers highlighted the relevance of the paper. However, some points were raised mainly about data presentation and discussion. Please see for details the reviewer comments below. In addition, the amount of figures is quite high. While you revise the data presentation - it would be great to check whether some figures could be merged or some data could go to the supplement.

Reviewer 1

In general, the authors provided a good amount of evidence to support their arguments. However, some of their data presentation and interpretations were confusing and needed to be significantly improved before publication. Please see detailed comments below.

1. The first common issue for this manuscript is that the main text sometimes refers the whole figure, instead of each individual figure panel, which makes the reader difficult to associate. For example, line 120, 174 etc. Please be specific to the relevant data panel.

2. The second common issue is that the authors tended to use the percentage (which is not really shown in the figure, but they were trying to compare the difference between groups) to interpret their findings. Meanwhile, they also have some percentage data (which is the y-axis in the figure). For example, line 185-188. It is really confusing throughout the manuscript.

3. Some of the organization and figure presentation are difficult to understand. For example, Fig.3 H-J; Fig.4 (no labeling to index the color for the images, the group labels are extremely small at the bottom. Same issue for Fig.5); Fig.5 G (which is not really discussed in that section but later); Fig.6 E (in the figure legend but not the actual figure); Fig.9 (no clear group labeling for the images).

4. For Fig.4, please provide the images for CC3 and colocalization images for Ki67 and PH3/ZIKV.

5. The method for RNAscope quantification is not clearly described and presented. From Fig.11, it is not easy to tell how they actually quantified those cells. Also Fig.11 F doesn't seem to have MFAP2 probe in there.

Reviewer 2

While some of the claims presented by the authors has been previously described (neural precursor selectivity, AXL as entry receptor for ZIKV), overall the manuscript offer important supporting evidence to previous findings and help in better understanding ZIKV infection in animal model at a molecular level.

Questions to the authors:

1) The authors use a model for their time course experiments in which ZIKV strain is injected into the lateral ventricle of prenatal mice in utero and the contralateral uterine horn is used for saline vehicle controls. The model allows to document that when fetuses are injected at E12.5, microencephaly is found at all examined time points. According to the data presented on Figure 3B, 3C, the differences in forebrain size show a consistent difference across all the time points. This indicates that the baseline of forebrain size was somewhat shifted (5-10% decrease, ∼100micron), but this difference did not increase, rather decrease over time between Mock and ZIKV infected. Surprisingly, ZIKV-induced decrease in brain size was not dependent on the age of infection. Overall, these set of experiment suggest that there is an immediate 100 micron decrease in gross brain size following ZIKV infection due to cell death and decreased proliferation, which does not impact further brain size. This observation brings up the question whether the immediate cell death and slow in proliferation rate after ZIKV infection is only temporary in early infections and proliferation rate returns to normal within a day or even compensate to some extent for the loss of NPCs? However, according to Figure 4A, cell death become elevated in the infected animals. Considering previous research indicating that mitosis is impaired during infection, can the authors comment on why the initial 100 micron difference does not further increase, especially when fetuses are injected at E12.5?

2) Findings regarding that aRGCs are targeted by ZIKV early in infection and that proliferation of this essential NPC class is reduced is convincingly demonstrated. Previous studies (e.g. Miner et al., 2016, Meertens et al., 2017) have already proposed that ZIKV gains entry into aRGC of the developing cerebral cortex by binding to the Axl receptor present on the surface of the aRGCs. At the same time, Wang et al (Journal of General Virology, 2017) suggested that no significant difference has been found between Axl-deficient mice and their littermates in terms of the survival rate, clinical manifestations, viral load, ZIKV distribution and histopathological changes in major organs. If that is correct, more than one receptor is possible to occur for ZIKV, which would also compromise the impact of Axl on ZIKV infection in mice. Authors should add discussion to better interpret their findings in the context of the existing literature.

3) Probably one of the most important and surprising finding is that ZIKV-induced decrease in brain size was not dependent on the age of infection. More discussion on this would be needed. It remains unclear if early infection has more severe consequences in any way to brain function. How does the ratio of aRGC1 and aRGC2 changes along between E12.5 and E15.5? Does Axl expression level changes, indicating that despite the findings, animals would be more vulnerable for infection at a certain stage? Taking into account that aRGC1 (Prc1+) may be fated towards direct neurogenesis while aRGC2 (MFAP2+) cells are primed towards the Tbr2+ bIPC lineage, what the authors think about a virus induced imbalance between the two cell population? Is it possible that early disturbance would induce some cell fate switch between the subpopulations?

4) Along with the previous points on the lack of sufficient discussion, I personally feel that relevance of the model in other virus studies is not needed to be discussed here, as it has very loose connection to the actual findings and the overall goal of the study. Hopefully the authors can indeed leverage on their model to better understand the impact of COVID and publish a separate paper.
